# Preliminary Study for the Preparation of Transmucosal or Transdermal Patches with Acyclovir and Lidocaine

**DOI:** 10.3390/polym13203596

**Published:** 2021-10-19

**Authors:** Cristina-Adela Marioane, Mădălin Bunoiu, Mădălina Mateescu, Paula Sfîrloagă, Gabriela Vlase, Titus Vlase

**Affiliations:** 1 Research Centre for Thermal Analysis in Environmental Problems, West University of Timisoara, Pestalozzi Street 16, 300115 Timisoara, Romania; cmarioane@yahoo.com (C.-A.M.); madalina.mateescu@e-uvt.ro (M.M.); titus.vlase@e-uvt.ro (T.V.); 2 Faculty of Physics, West University of Timisoara, V. Parvan Ave., No. 4, 300223 Timisoara, Romania; madalin.bunoiu@e-uvt.ro; 3 National Institute for Research and Development in Electrochemistry and Condensed Matter, Dr. A. Paunescu Podeanu Street, No. 144, 300569 Timisoara, Romania; paulasfirloaga@gmail.com

**Keywords:** alginate, lidocaine, acyclovir, PVP, PVA, FTIR, UV-Vis analysis, thermal analysis, drug delivery, membranes

## Abstract

The present study aimed to prepare and evaluate patches for the controlled release of lidocaine/acyclovir and the binary mixture between lidocaine: acyclovir in the oral cavity. Mucoside adhesive patches containing 12.5 mg/cm^2^ lidocaine/acyclovir or binary mixture base were developed by a solvent casting method using sodium alginate, polyvinylpyrrolidone (PVP), glycerol (Gly), polyvinyl alcohol (PVA), and Span 80 (S). Binary mixtures between all components were prepared before the patches’ formulation in order to be able to check the substance compatibility. All formulated patches were analyzed by FT-IR spectroscopy, UV-Vis analysis, thermogravimetry (TGA), and scanning electron microscopy (SEM). FT-IR and TGA analyses were also used to check compatibility between binary mixtures. The study establishes which membranes are indicated in the controlled release of lidocaine/acyclovir and those membranes that contain both active principles. Membranes based on alginate, PVP, and PVA can be used to release the active substance. Simultaneously, membranes with SPAN used as a gelling agent were excluded due to the interaction with the active substance. The following membranes composition have been chosen for lidocaine release: Alginate:Gly and Alginate:Gly:PVP. At the same time, the following membrane compositions were chosen for acyclovir membranes: Alginate:Gly:PVP and Alginate:PVA:Gly. Both active substances could be included to obtain a homogeneous distribution only in the membrane based on alginate, PVA, and Gly.

## 1. Introduction

The importance of transmucosal or transdermal patches has been shown over the years into handling and administering drugs [1]. Transdermal, transmucosal patches offer many advantages compared to conventional pharmaceutical formulations. The most important advantages are maintaining a constant and prolonged plasma level of the drug, reducing side effects, consistent drug release profile, etc. [2].

Transdermal delivery of drugs showed an impressive ascension in the last few decades due to advantages such as the absence of hepatic metabolism, gastric irritation and enzyme degradation [3].

Lidocaine (2-(diethylamino)-*N*-(2,6-dimethyl phenyl)-acetamide) (**L**) was discovered in 1948 and appeared in literature in 1958, being published by Clive-Lowe et al. [4], it is used generally in many clinical applications and at the same time is the most-used local anesthetic, being recognized for the intermediate duration of action [5,6] and class I b antiarrhythmic agent [7]. The usage of lidocaine was also considered as a treatment for acute tinnitus in the last period [8]. It is the monocarboxylic acid amide obtained from the formal condensation of *N*,*N*-diethylglycine with 2,6-dimethylaniline. It is a monocarboxylic acid amide, a tertiary amino compound, and a member of benzenes. It derives from a glycinamide [9].

**L** is recognized for its ability to block reversible neuronal conduction of noxious stimuli. Blocking occurs by binding to the voltage-gated sodium channel with the activity of the excitable membranes [6]. The **L** is 60–80% protein-bound during intravenous administration, primarily to α-1-acidic glycoprotein [10]. **L** crosses the blood–brain barrier through the process of passive diffusion through membranes. Recent studies also demonstrated that L exhibits bactericidal effects against *E. coli*, *S. sanguinis*, and *S. salivarius*. The effectiveness of this effect is determined by the exposure time [11]. **L** may exist in two forms as ionized or unionized. It is soluble in water and very soluble in alcohol, chloroform, and benzene [12].

Acyclovir (**Av**) or 9-[(2-hydroxyethoxy) 62 methyl] guanine is an acyclic nucleoside analog that was first approved for use in 1982. It is an oxopurine that is guanine-substituted at position nine by (2-hydroxyethoxy)methyl substituent. **Av** is a synthetic analog of purine nucleoside and presents essential antiviral activity against viruses. Av also competitively inhibits viral DNA polymerase by incorporating it into the growing viral DNA chain and terminating further polymerization after conversion to the active metabolite acyclovir triphosphate. This conversion takes place by viral thymidine kinase [13]. **Av** is slightly soluble in water, with a maximum solubility of 2.5 g/L at physiological pH, soluble in dilute aqueous solutions of alkali hydroxides and mineral acids [14], and is being used to treat infections such as herpes simplex virus [15], cutaneous herpes, genital herpes, varicella-zoster infections to treat HSV encephalitis, and neonatal HSV infections, etc. [16,17,18] The ways the antiviral agents may act include: preventing viral replication this can be done by inhibiting viral DNA polymerase; blocking late stages of virus assembly; binding to specific cell-surface receptors; inhibiting viral protein synthesis or inhibiting viral penetration or uncoating [19]. Av can be found on the current market in different forms, being available as 200, 400, and 800 mg tablets; 500 or 1000 mg lyophilized powder for intravenous injection; 200 mg/5 mL suspension; 3% ophthalmic ointment in petrolatum; a 5% *w*/*w* cream in a miscible water base, etc. [20]. Topical **Av** prescription is recommended for initial episodes of herpes simplex virus infections. At the same time, this is recommended to treat limited non-life-threatening mucocutaneous herpes simplex virus (HSV-1 and HSV-2) infections in immunocompromised patients.

Nevertheless, systemic Acyclovir may be preferred because it is more efficient [21]. **Av** absorption from the gastrointestinal tract is variable and incomplete. After absorption, it is generally distributed into body tissues and fluids, including the brain, muscle, spleen, saliva, lung, liver, kidney, uterus, vaginal mucosa, cerebrospinal fluid herpetic vesicular fluid, and placenta [22].

The oral absorption of acyclovir is dose-dependent and with low bioavailability. Percutaneous absorption of acyclovir is low. The drug is well tolerated on oral application, but in case of intravenous administration, sweating and rashes are observed. Topical application of drug may cause burning and stinging sensations [23].

Therefore, there is a need to formulate patches with acyclovir and an anesthetic compound that promotes an appropriate balance between effective release and skin safety. Co-administration of lidocaine with acyclovir helps to relieve the pain associated with cases of Oral Herpes, Herpetiform Stomatitis, or Recurrent Herpetiform Aphthous Ulcers.

Transdermal patches offer the possibility of a single administration of the required dose over an extended period of time, which leads to a simplified medication regimen. According to studies, it has been observed that drugs with low bioavailability are suitable for a transdermal pathway.

This study aims to formulate patches with different components, containing lidocaine and acyclovir drugs. These patches should act as a transmucosal drug delivery system. The aim of this system is to act as an analgesic and antiviral agent that can be used in case of Oral Herpes, Herpetiform Stomatitis, or Recurrent Herpetiform Aphthous Ulcers [24].

During the obtaining and developing of new pharmaceutical formulation, it is very important to know the physico-chemical properties of drugs and pharmaceutical components. Current pharmaceutical laws oblige drug manufacturers to assess the compatibility of active substances, excipients, and drugs. Thus, the characterization of the active pharmaceutical ingredients and excipients leads to the improvement of the quality parameters of all the raw materials used during the manufacturing process of pharmaceutical products, as well as those of the final products [25].

Literature proves that thermal analysis techniques are methods used in the physico-chemical characterization of various materials used in the pharmaceutical industry. Thermal analysis techniques such as differential scanning calorimetry (DSC), differential thermal analysis (DTA), and Thermogravimetry (TG) are widely used in studies of pre-formulation, drugs, and substance development of pharmaceutical interest [26,27]. Thermal analysis techniques are used in many studies to study incompatibilities between active substances: excipients [28,29,30,31] and also Pharmacopeias described the thermal analysis methods as general techniques. However, at the same time, the description of their uses is relatively limited on Pharmacopeias because only a few studies on purity determination, loss on drying of reference substances, polymorphic studies, solvates and hydrates, determination of melting point, and quantification of volatile components in drugs are highlighted in Pharmacopeias. The use of thermal analysis techniques in the pharmaceutical field has taken a break lately, adding the results of other complementary studies such as FTIR, UV-Vis, RX, and others [32].

According to literature research to date, no studies have been performed on transdermal patches with acyclovir and lidocaine. These encouraging outcomes of study warrant further investigation to examine the efficacy of these patches in the treatment of Oral Herpes, Herpetiform Stomatitis, or Recurrent Herpetiform Aphthous Ulcers infections.

The present study aims to obtain the best alginate-based membrane pharmaceutical formulation containing antiviral embedded Acyclovir and anesthetic and antimicrobial lidocaine. Thus, the pharmaceutical formulation will have both benefits from the administration of the active principles and benefits related to its complex biological activity, namely antiviral, antifungal, and anesthetic. The current pharmaceutical formulations used for topical administration of Acyclovir are gels that last for a limited time in the oral mucosa. Therefore, the new formulation in the form of a mucoadhesive membrane would thus succeed in maintaining both active principles for a more extended period. The presented study manages to establish by combining the results of several complementary physico-chemical techniques (TG, FTIR, SEM-scanning electron microscopy, UV-Vis ultraviolet-visible), which would be the best membrane variant that can be used to release the active substances studied both individually as well as in the mixture form.

## 2. Materials and Methods

**Figure 1:**
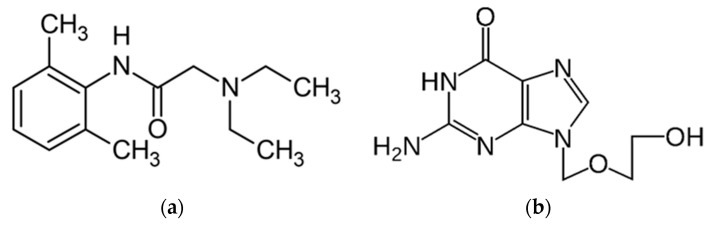
(**a**) Structure of Lidocaine **L** (SA); (**b**) structure of Aciclovir **Av** (SA).

Alginate from Sigma-Aldrich P.N., (Saint Louis, MO, USA, W201502), Polyvinylpyrrolidone, M.W. 40,000 powder (Calbiochem, Merck, Darmstadt, Germany, Lot: BCBV6638, CAS: 9003-39-8), Glicerin-ChimicReactiv SRL, Span 80-S (Sigma-Aldrich, St. Louis, MO, USA, PC-1002614428 S6760-250 mL, Lot # MKCF4138)-nonionic surfactant, Polyvinyl alcohol, (Merck, Darmstadt, Germany, S7316066 641). The active substance (SA), Lidocaine hydrochloride L-(SA) (Figure 1a) purchased from USP standards and acyclovir Av-(SA) (Figure 1b) purchased from Sigma-Aldrich.

For the compatibility studies of the active substances with the membranes, it was considered to follow the processes that take place at membrane synthesis. In this study, the presence of active substances in the synthesized membranes was followed and thus it was desired that the active substances suffer the same stages as those present in the membrane, being aware that the active substances will not necessarily be found in the initial crystalline forms. Therefore, in the first part of the article, the active substances in the initial form (noted SA) were compared by FTIR and thermal analysis with the moistened and then dried ones (noted D).

### 2.1. Patches Preparation

The sodium alginate solution was prepared by dissolving sodium alginate in water (10 g/L) for 2 h using a magnetic stirrer, at room temperature, and the volume corresponding to each formulation was used to prepare the patch. PVP and PVA polymers were dissolved separately before preparation into a mixture of IPA: acetone in a ratio 1.5:1. Glycerol and Span 80 was added as a plasticizer and surfactant. After preparation, the membranes were poured into crystallizers, kept at room temperature for 48 h and stored in polyethylene packaging until analysis. The quantities of components used in membrane synthesis are shown in Table 1. After three days, the patches were visually inspected and analyzed.

### 2.2. FTIR-UATR Spectra

FTIR-UATR spectroscopy data were collected using a Perkin Elmer SPECTRUM 100 device and employing the Universal Attenuated Total Reflectance (UATR) technique. Data collection was performed after 8 consecutive recordings at a resolution of 4 cm^−1^ on the spectral range 4000–650 cm^−1^.

### 2.3. Thermogravimetric Analysis

The thermal behavior was determined using aluminum crucible on TG/DTA Diamond Thermal Analyzer produced by Perkin Elmer. Analyses were performed in a dynamic air atmosphere (synthetic air 5.0 Linde Gas with flow 100 mL∙min^−1^) at a heating rate, β = 10 °C·min^−1^, in temperature range 30–400 °C for patches and in temperature range 30–500 °C for active substances. Studies were performed on the pure substances and on the patches. All analyzed samples had a mass between 8 and 12 mg.

### 2.4. SEM Analysis

An Inspect S scanning electron microscope was used to record the images of the microstructure of the membranes’ surfaces. The analysis was carried out in low-vacuum conditions (60 Pa) at an accelerating voltage of 25 kV. Due to the fact that the studied membranes are non-conductive, for morphological analysis of the surface a Large Field Detector (LFD) was used. For a comparative study, the microstructure of the studied materials was recorded at a magnification of 600 (100 μm).

### 2.5. UV-Vis Spectrophotometry

The UV-Vis spectra were obtained using T90^+^ UV-Vis Spectrophotometer with double beam in photometric range: 190–450 nm. All absorbance measurements were taken in a 10 mm UV/Vis spectroscopy cell at room temperature, using distilled water as a blank. Studies was performed for substance active standard and for mucoadhesive membrane. The studies were performed for the standards obtained by solubilizing the active substances and for the mucoadhesive membranes characterized by SEM and validated by the other techniques. The studies were performed for qualitative purposes.

## 3. Results and Discussion

### 3.1. FT-IR Spectra

#### 3.1.1. FT-IR Spectra of the Active Substances

**Figure 2:**
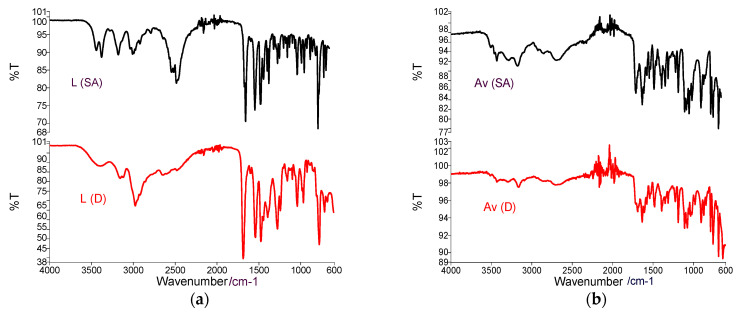
(**a**) FTIR spectra of Lidocaine **L** (SA))/Lidocaine dissolved **L** (D); (**b**) FTIR spectra of Acyclovir **Av** (SA) and Acyclovir **Av** (D).

During the FTIR analysis of the active substances, the following specific bands were observed (Figure 2a—Lidocaine **L** (SA)/**L** (D)), Figure 2b—Acyclovir **Av** (SA)/**Av** (D). The FTIR spectrum of the active substances was designed to compare with the spectra of medicated patches. The spectrum of active substances presents the characteristic bands according to the literature [33].

The FTIR spectrum of Lidocaine (SA) and Lidocaine (D) shows vibrations at 3500 cm^−1^ related to the –NH group as well as the presence of intermolecular H bonds), Vibrations at 3100–3000 cm^−1^ specific to the CH bond in aromatic compounds and the C=H bond at 1750 cm^−1^ argues the presence of the CO bond within the carbonyl groups, at 1680 cm^−1^ the vibration of the NH bond is present in quinolones, at 1550 cm^−1^ the presence of the extended carbonyl group CO is argued; at 1250 cm^−1^ OH delta and at 1050 cm^−1^ [32]. For the **L** (D), the bands from the region 3500–2500 cm^−1^ present a higher intensity, and the bands from the region 1750–1250 cm^−1^ appear a little bit earlier. These changes can be argued by the modification of the substance from the crystalline phase into the amorphous phase.

The FTIR spectrum of **Av** (SA)/**Av** (D) shows a characteristic O–H vibration region with two peaks at 3434 and 3398 cm^−1^. The peaks for primary amines can be observed at 3280 and 3160 cm^−1^. At the same time, the peak characteristic of the secondary amines is clearly visible at 3088 cm^−1^. The peak at 1630 cm^−1^ represents an N–H bond of primary amines and the peak visible at 1708 cm^−1^ are attributed of the stretching vibration of the –C=O group, 1478 cm^−1^ vibration for C=N group, at 1182 cm^−1^ characteristic for C–N. The appearance of the bands in the region of the wave number 2000–1650 cm^−1^ and the CH extension region of the FTIR spectrum over 3000 cm^−1^ indicate the presence of an aromatic group. For **Av** (D), intensity bands are lower than in the initial active substance, which can be argued as in the case of lidocaine by modifying the crystalline form of the substance. The first stage of the studies is considered a study of the compatibility between the active principles and the components in the membranes. The study aims to identify possible interactions between these components.

#### 3.1.2. FTIR Study of Binary Mixtures–Active Substances: Patch Components

**Figure 3:**
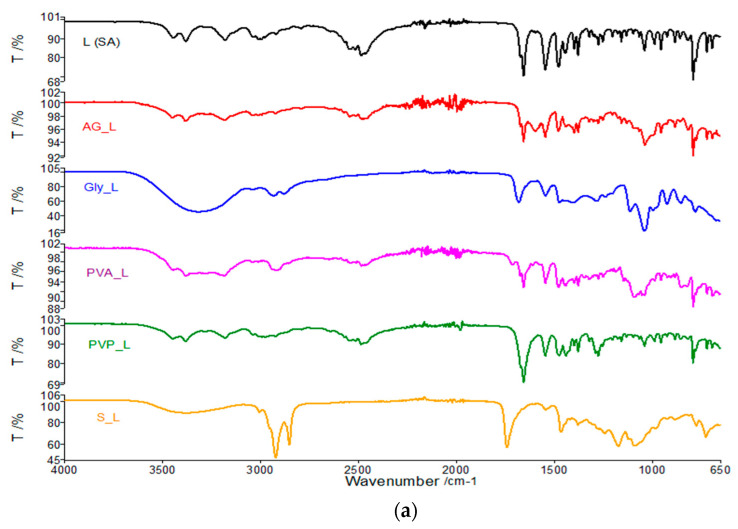

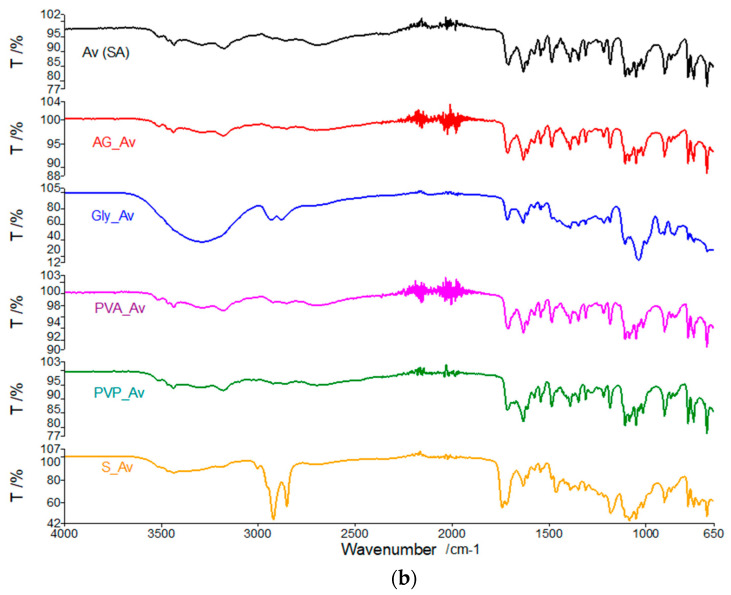
(**a**) FTIR spectra of **L** (SA) compared to binary mixtures of the active substance with the components used in membrane synthesis (1:1); (**b**) FTIR spectra of **Av** (SA) compared to binary mixtures of the active substance with the components used in membrane synthesis (1:1).

To identify possible interactions between the reactants and active substances proposed for patch preparation, we decide to prepare a binary mixture between each component and the active substances. The binary mixtures were obtained by mechanical mixing of the components in a mass ratio of 1:1. These mixtures were analyzed by FTIR spectroscopy and thermal analysis and the results were compared with the results obtained by the same techniques in the case of the active substances **L** (SA) (Figure 3a) and **Av** (SA) (Figure 3b).

For the binary mixtures obtained between **L** (SA) and membrane components, it can be observed that for the Gly-**L** (SA) mixture spectrum, the characteristic bands of the active substance are not highlighted very well, these being covered by the Gly spectrum. In the case of the binary mixture obtained between Span and **L** (SA), there are a lack of bands from 1650 cm^−1^ related to the CO bond within the carbonyl group. Therefore, a reaction of the Span component with the active substance at the carbonyl bond may be suspected. For the rest of the binary mixtures, the bands characteristic to the active substance can be observed.

Analyzing these figures can be seen very clearly in addition to the bands characteristic of the components used in membrane synthesis and peaks characteristic to the active substance **Av** (SA). This statement argues that the active substance **Av** (SA) does not interact with membrane components while in the case of **L** (SA), and can be considered a possible interaction with Span.

#### 3.1.3. FTIR Study of Synthesized Membranes

Within the membranes, the obtained spectra will be compared with the active substances **L** (D) and **Av** (D). In order to validate the presence of the active substance Av in the membrane, the main absorption peaks at 3500–3400 cm^−1^ (NH), 1695 cm^−1^ (C=O), 3282 cm^−1^ (NH_2_), 1478 cm^−1^ (C=N) and 1182 cm^−1^ (C–N) and in the case of the L substance the presence of peaks from 3400 cm^−1^ specific NH bond stretches will be followed, vibration from 3100 cm^−1^ (CH), 1680 cm^−1^ C=O characteristic for amide I, at 1470 cm^−1^ (CN) amide II and at 960 cm^−1^ Fingerprint area. It can be easily seen that the bands characteristic of the two active substances are located in the same spectral areas [34,35].

**Figure 4:**
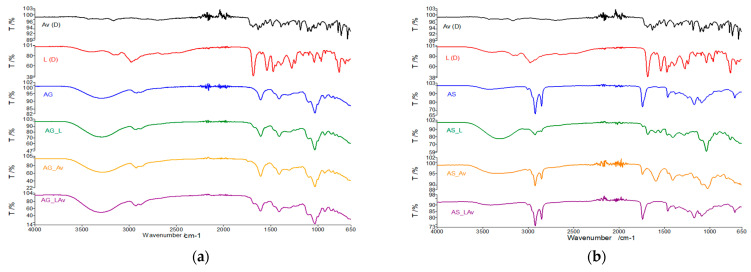

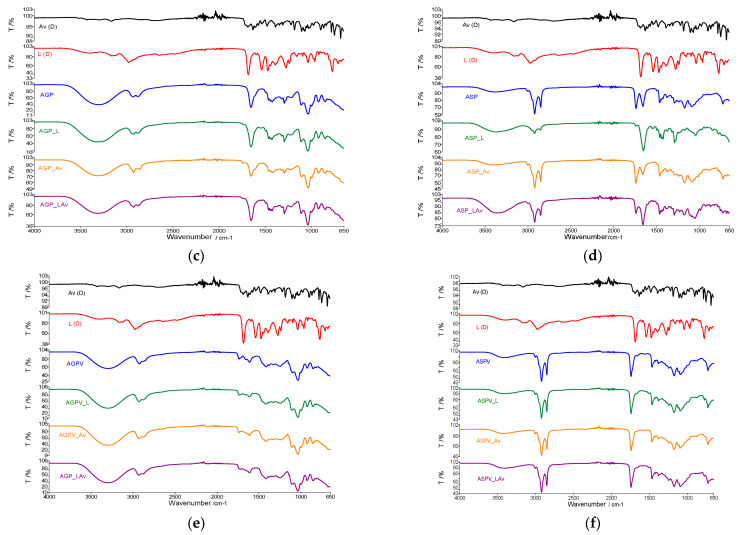
(**a**) Comparative FTIR spectra of **Av** and **L** with the membranes spectra based on alginate and Glycerol; (**b**) Comparative FTIR spectra of **Av** and **L** with the membranes spectra based on alginate and Span; (**c**) Comparative FTIR spectra of **Av** and **L** with the membranes spectra based on alginate, Gly and PVP; (**d**) Comparative FTIR spectra of **Av** and **L** with the membranes spectra based on alginate, Span and PVP; (**e**) Comparative FTIR spectra of **Av** and **L** with the membranes spectra based on alginate, Gly and PVA; (**f**) Comparative FTIR spectra of **Av** and **L** with the membranes spectra based on alginate, Span and PVA.

Analyzing the overlapping curves of **Av** and **L** with the membranes’ spectra based on alginate and glycerol (Figure 4a), we can say that both individual and mixed membranes with lidocaine and acyclovir present the representative peaks of acyclovir, but these are easily visible in the FTIR spectrum of membranes. Here, it is essential to mention that the characteristic bands of the membranes partially cover the distinctive bands of the active substances.

If Span was used for synthesis of Alginate membranes (Figure 4b), the characteristic Av peaks are easily covered in the membrane with a single active principle, and of course the same thing can be observed for the membranes with both active substances.

Regarding the active substance **L**, it is very well visible in the membrane with a single active principle by clearly highlighting bands from 2950 cm^−1^, 1680 cm^−1^, 1470 cm^−1^, from 960 cm^−1^ and slightly displaced for the higher wave lands. In the case of the membrane with both active principles, the bands from 1680 cm^−1^ shifted to 1740 cm^−1^ are much better highlighted, the bands specific to the C=N (1470 cm^−1^), and 1180 cm^−1^ are clearly visible, this one is specific to C–N bond from acyclovir, and fingerprint specific for lidocaine to 960 cm^−1^. We can assume an interaction at the level of the C=O group within the active substances.

The spectral analysis of the recrystallized active substances and alginate, glycerol and PVP membranes all peak characteristics for L are highlighted very well for all membranes with the individual active substance (Figure 4c), The bands are better highlighted in the membrane with lidocaine because the concentration of the active substance is higher in this case. The same conclusions emerge in the case of the active substance **Av**.

The FTIR spectra of membranes based on alginate, PVP, and Span (Figure 4d), highlight the characteristic peaks of active substances much more clearly [36]. It seems that in this membrane variant, Span does not show interactions with **L** (or **Av**). This highlighting of the active substance groups is because, for this membrane, the amount of gelling agent is smaller than in the studies performed on binary mixtures. Otherwise, we can conclude that in this type of preparation, the characteristic groups for Span are blocked by polymerization.

In the membranes based on alginate, glycerol, and PVA (Figure 4e), the FTIR spectrum of the membranes with the active principle highlights the characteristic peaks of the active substances. Indeed, they are less represented by the spectrum of active substances. This conclusion can be argued by the fact that the concentration of active substances in membranes was lower and by the fact that the characteristic bands of membranes largely cover the FTIR bands of **L** and **Av**.

The characteristic bands of the active substances in membranes based on PVA, Span, and alginate overlap with the FTIR spectrum of the membrane without the active principle (Figure 4f). Therefore, we cannot highlight by spectroscopic analysis the presence of active substances in membranes, and the results that occurred after thermal analysis will provide more information. In the case of all studied membranes, they will resort to the comparative analysis of recrystallized active substances, of membranes without active substances, and of active membranes with one or both active substances.

### 3.2. Thermogravimetric Analysis

**Figure 5:**
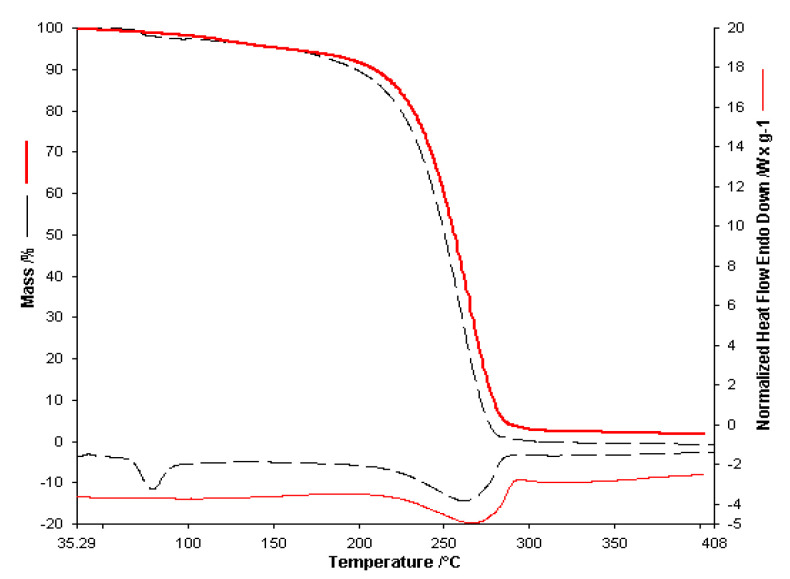
The thermoanalytical curves of **L** (SA)-- - - and **L** (D)___ obtained with a heating rate of 10 °C∙min^−1^.

**Figure 6:**
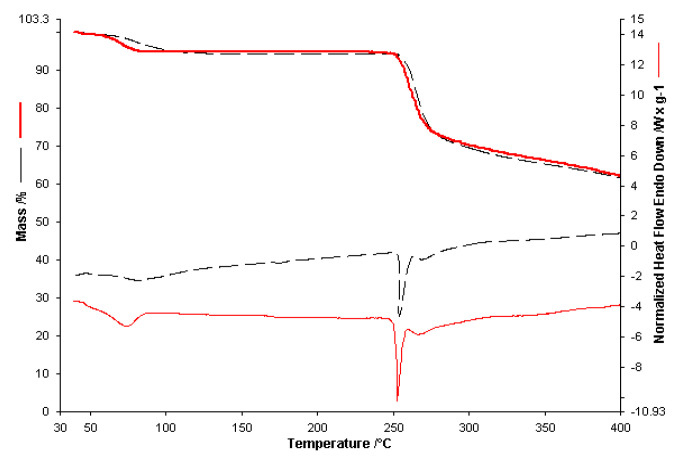
The thermoanalytical curves of **Av** (SA)___ and **Av** (D)____ obtained with a heating rate of 10 °C∙min^−1^.

The same experimental protocol as in the spectroscopic study was used in the case of thermal analysis. Thermal analysis begins with the thermogravimetric study of the active substances in the initial form **L** (SA) and **Av** (SA) and compared with the results of the analyses obtained in the case of **L** (D) and **Av** (D). These results will be used as a benchmark of these substances in membranes, taking into account the fact that we have different concentrations. The thermal analysis of the active substances (SA) and of the wet and then dry (D) active substances are presented comparatively in Figure 5 for **L** and in Figure 6 for **Av**.

The thermal analysis performed in the case of **L** (SA) in the air atmosphere with a heating speed of 10 °C/ min up to 400 °C highlighted on the TG curve two decomposition processes one in the range 70–150 °C with a maximum loss 5% of the initial mass of the sample due to evaporation of volatile compounds, respectively a second decomposition process that leads to the total loss of mass that takes place in the range 160–290 °C. On the HF curve, an endothermic process with a maximum of 79 °C can be highlighted, which can be attributed to the melting process of the sample. A single endothermic process, seen at 262 °C, can be seen for the second stage. Thermal analysis of the **L** (D) active substance does not show the stage for the 70–150 °C interval. The decomposition for the 160–290 °C stage is similar to the one for the **L** (SA) sample.

The thermal analysis performed for **Av** (D) takes place in the same conditions. It presents two processes, namely the first endothermic process in the range 40–130 °C with a mass loss of about 6% of the sample mass. The second process takes place through a decomposition stage in the range 252–300 °C. On the HF curve, two delimited endothermic processes were observed. The first maximum can be attributed to the melting process of the sample (260 °C), followed by decomposition. Considering the structure of the active substance, we can say that this process could be attributed to the thermal decomposition of the drug and may correspond to cleavage. This cleavage is followed by removing the alcohol-ether side chain from the acyclovir structure and the consequent formation of guanine as a residual mass [33]. The mass loss in this range is 24% of the sample mass with a maximum on the DTG curve at 270 °C.

Comparing the overlapped curves for empty and active substance membranes, along with thermograms for **L** and **Av** active substances, shows that active substance membrane thermal decomposition shows the active substance contributions, both in thermal effects and share of decomposition stages. Contrary to expectations, the melting process for active substances is not visible.

Membranes with Span as a gelling agent, as seen in Table 2, show a highly exothermal decomposition stage at 300 °C. For active substance membranes, this stage is observed at higher temperatures. A simple preliminary visual inspection, supported by the FTIR results for the binary mixes and membranes, show that membranes with Span as a gelling agent are brittle. FTIR analysis of the binary mixes indicate a possible interaction with Span in the case of active substance **L**. As such, only the thermogravimetric results for membranes with glycerol as a gelling agent, as it produces homogenous and non-brittle membranes, were graphically represented and included here in the presentation of the results.

**Figure 7:**
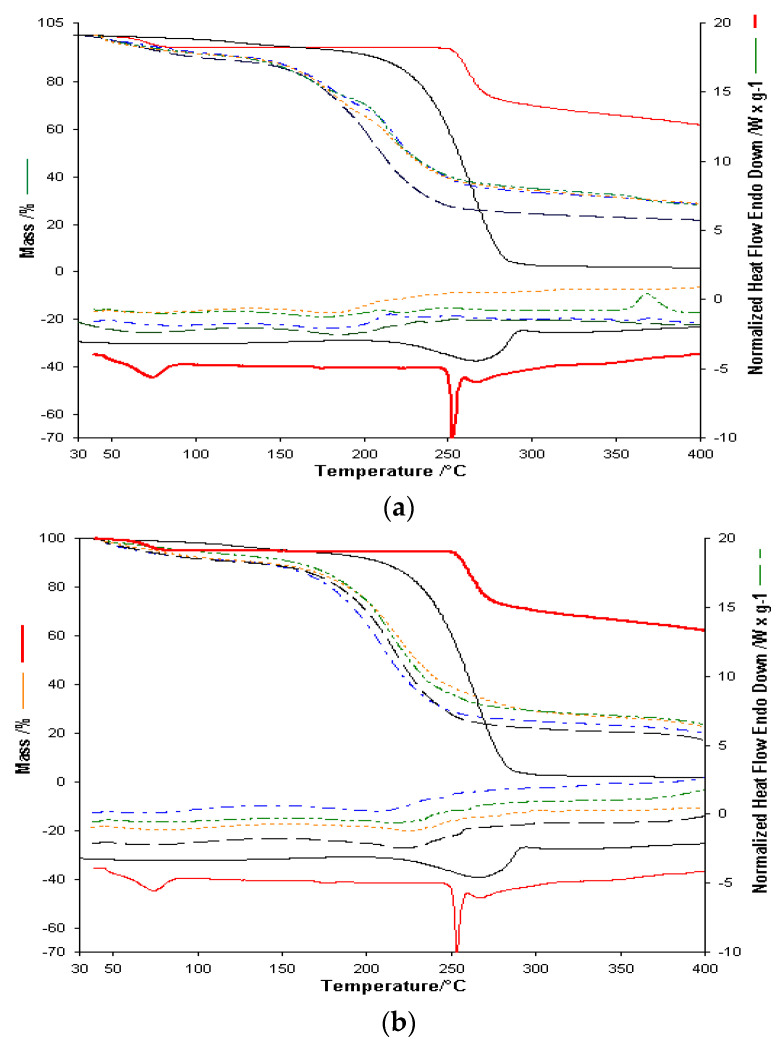

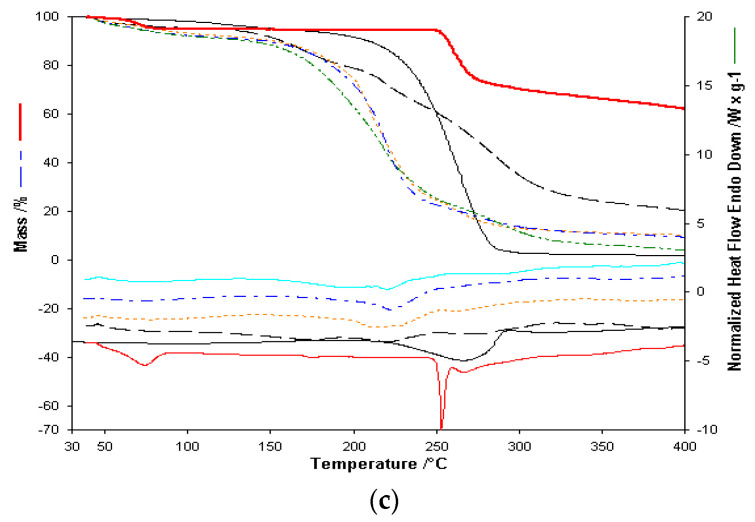
(**a**). Comparative of TGA and HF for Av(D)___, L(D) ___, AG_ _, AG_Av…., AGL_ ._ ., AGLAv .. _ .. _.; (**b**). Comparative of TGA and HF for Av(D) ___, L(D) ___, AGP - - -, AGP_Av…., AGPL_ ._ , AGPLAv .. _ .; (**c**). Comparative of TGA and HF for Av(D)____, L(D) __, AGPV - - -, AGPV_Av……., AGPVL_ ._ , AGPVLAv .. _ ...

In order to analyze the possible interactions between the membrane and the active substance, the results for both the single active substance and two active substances membranes, in comparison with non-active substances membranes, will be shown in the following (Figure 7). Considering the fact that active substances are present in smaller quantities in active substance membranes, the focus will be on the thermal effects specific to the decomposition stages of active substances and the thermal effects for the membrane decomposition.

For alginate-based membranes with glycerol as a gelling agent, decomposition stages for active substances within are observable, though at a lower temperature, explained by the distribution of the active substances within the membranes. Considering that active substance membranes have the same concentration of active substances, and the two active substances have a single distinct decomposition stage over 250 °C, we can determine similar thermal behavior for single active substance membranes. As expected, no active substance melting processes are observed. For two active substance membranes, considering the lower concentration of each active substance, the endothermal decomposition process, seen previously over 500 °C [27], occurs at 360–380 °C, which can be explained by the Av distribution and concentration within the membrane, thus indicating a possible interaction with said membrane. For membranes containing only **Av**, the active substance concentration is double, and therefore this process is apparent at temperatures higher than 400 °C.

At first glance, thermal analysis for alginate, PVP and Gly based membranes shows similar thermal behavior. Decomposition of active substances is observed to occur at lower temperatures for both active substances. The exothermal process for the decomposition of Av starts at 380 °C. The main decomposition stage for both substances is observed on the Heat–Flow curve at 260 °C. The thermal decomposition for the membrane with both active substances is similar to the one seen for the single active substance membrane, which indicates different destabilization of the active substances.

Thermal analysis for alginate, PVA, and Gly based membranes showed a different thermal behavior for active substance membranes in comparison with inactive-based ones. The TG curve shows a similar mass loss for membranes with one active substance, which is supported by the different destabilization also seen in the cases of the previous membranes and the two active substances **Av** and **L**. The two active substances membrane shows different thermal behavior, with the decomposition stages occurring at slightly different temperatures, though in similar percentages due to the same quantity. The endothermic process is no longer visible, as it occurs at higher temperatures, which indicates that both active substances are intact within the membrane.

The FTIR spectroscopy and thermoanalytical studies have led to the following hypothesis:

1. The use of glycerol as a gelling agent is recommended, as it can avoid interactions with **L** in small quantities.
2. Glycerol is useful in obtaining homogenous and non-brittle membranes.
3. Regarding alginate-based, alginate and PVP based, and alginate and PVA based membranes, these can be utilized to enable the release of each active substance in membranes with a single active substance [37].
4. For membranes that enable the release of both active substances, based on the obtained results, we can only recommend the homogenous and non-brittle alginate, PVA and glycerol-based membrane with both active substances visible.

Following the partial conclusions presented, SEM analysis was conducted only for those membranes which yielded favorable results with regards to aspect and homogeneity, as well as FTIR and thermogravimetric results. Therefore, below are images obtained through SEM analysis for alginate and Gly with **L**-based membrane, on alginate, PVP, and Gly with **L** and **Av** with a single active substance/based membrane, as well as alginate, PVA, and Gly in both variants (the single active substance and with both active substances) based membranes.

### 3.3. SEM Analysis

The studied membranes were analyzed in low vacuum, at 600× magnification.

**Figure 8:**
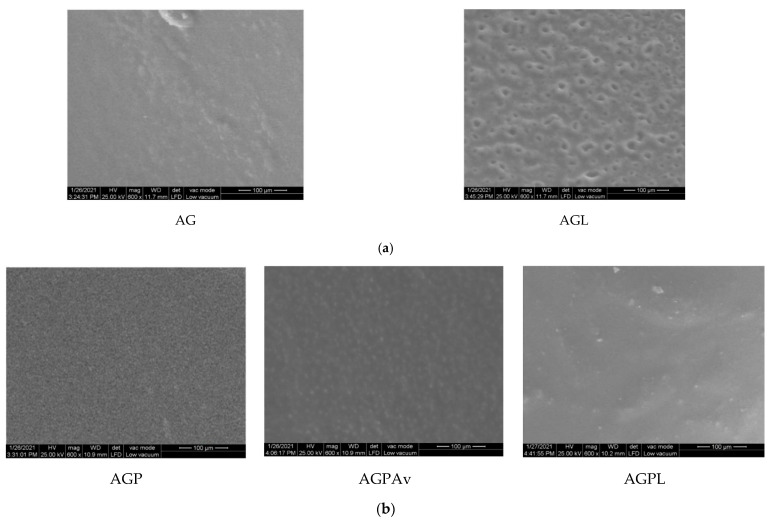

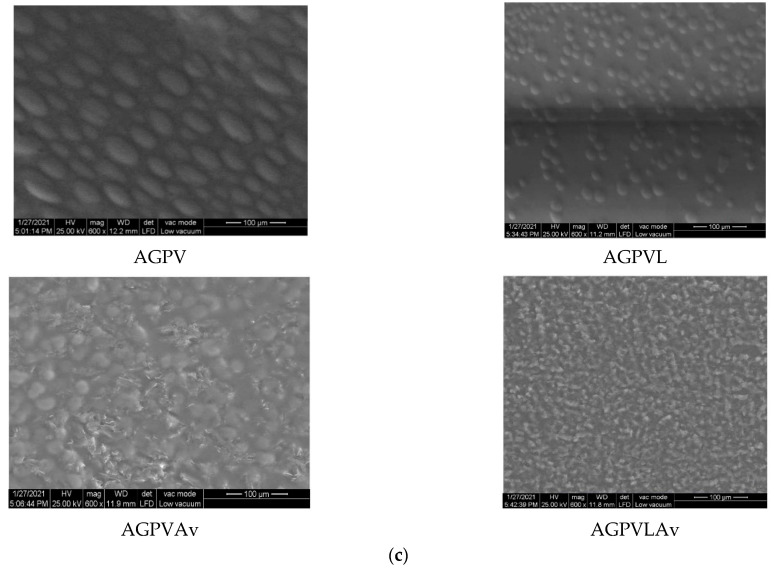
(**a**) SEM analysis for validated membranes based on alginate and Gly and active substances. (**b**) SEM analysis for validated membranes based on alginate, PVP and Gly and active substances. (**c**) SEM analysis for validated membranes based on alginate, PVA and Gly and active substances.

Figure 8a shows that **L** has a heterogeneous morphological structure, because some larger parts, other smaller, and even some intermediate parts are observed, but the distribution is homogeneous. In the case of AGP membrane (Figure 8b), homogeneous membranes are observed with active substances visible within the membranes. Within the AGPV membrane (Figure 8c), the two basic components of the Alginate membrane and PVA are observed. Moreover, the homogeneous distribution of the active substances within the membranes is observed both in the variant with a single active substance and with both active substances.

### 3.4. UV-Vis Spectrophotometry

All absorbance measurements were taken in a 10 mm UV/Vis spectroscopy cell at room temperature, using distilled water as a blank. Studies were performed for the substance active standard and for the membrane. The concentration of the standards was 1 mg/mL. Regarding the membranes, the amount of membrane used was chosen so as to keep it around 1 mg/mL of active substance. The UV-Vis study carried out considers a qualitative evaluation of the membranes. Only the membranes validated by the other techniques, which were presented in the SEM study, were analyzed by UV-Vis technique.

**Figure 9:**
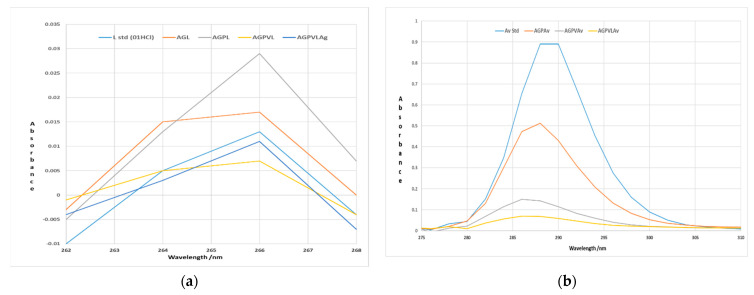
(**a**) Absorbance obtained in the case of membranes compared with the standard of the active substance **L**; (**b**) Absorbance obtained in the case of membranes compared with the standard of the active substance **Av**.

Figure 9a,b show the absorbance obtained in the case of the membranes studied compared to the standard of the active substance. **Av** and **L** were visible in all membranes having the maximum absorbance for **L** at 266 nm and membranes with Av content at 288 nm [38,39]. Following the analysis by UV-Vis spectroscopy, the presence of the active substance in the synthesized membranes is observed in all cases.

## 4. Conclusions

The study in this paper presents the synthesis and characterization of a larger number of alginate-based membranes in order to obtain the best membrane variant leading to transdermal release to the oral mucosa of lidocaine and acyclovir for the treatment of viral and microbial diseases in the mouth. In the membrane synthesis stage, alginate was chosen as a biopolymeric material individually or in a mixture with other polymers such as PVP and PVA. Binary mixtures between active substances and membrane components were prepared in order to be able to analyze biocompatibility between substances. Binary mixtures between all membranes’ components were analyzed using FTIR and thermogravimetric techniques.

Only homogeneous membranes whose components did not show interactions highlighted by FTIR and Thermal Analysis were analyzed by SEM microscopy to validate homogeneity. The presence and thus the availability of active substances was also supported by UV-Vis analyzes.

Studies have shown that the respective alginate-based membranes, PVP and PVA for the release of lidocaine and the alginate-based membranes, PVP and PVA for the release of acyclovir, are usable. A single membrane, namely one based on alginates and PVA, was considered to meet all the conditions to be used in order to release the mixture of active substances. SEM analysis argued the choice of homogeneous membranes. The clinical importance of the study can be underlined by the fact that membranes were obtained for active substances releases, both individually and in the mixture, which facilitates the treatment of diseases in the oral mucosa. Membranes are easily obtained and release active substances for a more extended period than current pharmaceuticals. Hence, we believe that they will lead to an application as soon as possible in vivo.

## Figures and Tables

**Table 1:** Patches formulation.

Ingredient	Av	L	Alginate	PVP	PVA	Gly	Span 80	Sample Aspect	Ingredient ID	Av	L	Alginate	PVP	PVA	Gly	Span 80	Sample Aspect
ID	mg	mg	mg	mg	mg	mL	mL			mg	mg	mg	mg	mg	mL	mL	
AG	-	-	50	-	-	0.3	-	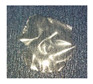	AGPL	-	12.5	25	25	-	0.3	-	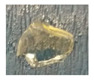
AS	-	-	50	-	-	-	0.3	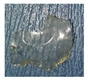	ASPL	-	12.5	25	25	-	-	0.3	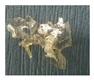
AGAv	12.5	-	50	-	-	0.3	-	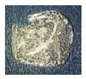	AGPLAv	6.25	6.25	25	25	-	0.3	-	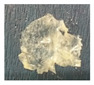
ASAv	12.5	-	50	-	-	-	0.3	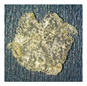	ASPLAv	6.25	6.25	25	25	-	-	0.3	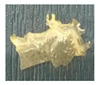
AGL	-	12.5	50	-	-	0.3	-	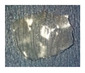	AGPV	-	-	25	-	25	0.3	-	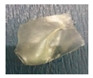
ASL	-	12.5	50	-	-	-	0.3	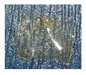	ASPV	-	-	25	-	25	-	0.3	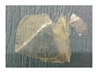
AGLAv	6.25	6.25	50	-	-	0.3	-	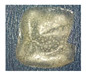	AGPVAv	12.5	-	25	-	25	0.3	-	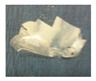
ASLAv	6.25	6.25	50	-	-	-	0.3	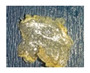	ASPVAv	12.5	-	25	-	25	-	0.3	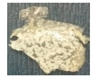
AGP	-	-	25	25	-	0.3	-	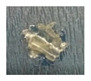	AGPVL	-	12.5	25	-	25	0.3	-	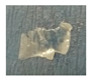
ASP	-	-	25	25	-	-	0.3	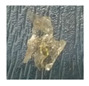	ASPVL	-	12.5	25	-	25	-	0.3	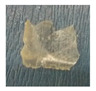
AGPAv	12.5	-	25	25	-	0.3	-	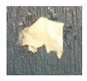	AGPVLAv	6.25	6.25	25	-	25	0.3	-	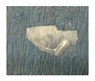
ASPAv	12.5	-	25	25	-	-	0.3	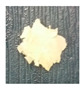	ASPVLAv	6.25	6.25	25	-	25	-	0.3	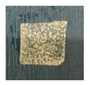

Membranes legend: AG—Alginate:Glycerol; AS—Alginate:Span; AGAv—Alginate:Glycerol:Acivlovir; ASAv—Alginate:Span:Aciclovir; AGL—Alginate:Glycerol:Lidocaine; ASL—Alginate: Span:Lidocaine; AGLAv—Alginate:Glycerol:Lidocaine:Acivlovir; ASLAv—Alginate:Span:Lidocaine:Aciclovir; AGP—Alginate:Glycerol:PVP; ASP—Alginate:Span:Glycerol; AGPAv—Alginate:Glycerol:PVP:Aciclovir; ASPAv—Alginate:Span:PVP:Aciclovir; AGPL—Alginate:Glycerol:PVP:Lidocaine; ASPL—Alginate:Span:PVP:Lidocaine; AGPLAv—Alginate:Glycerol: PVP:Lidocaine:Aciclovir; ASPLAv—Alginate:Span: PVP:Lidocaine:Aciclovir; AGPV—Alginate:Glycerol:PVA; ASPV—Alginate:Span:PVA; AGPVAv—Alginate:Glycerol:PVA:Aciclovir; ASPVAv—Alginate:Span:PVA:Aciclovir; AGPVL—Alginate:Glycerol:PVA:Lidocaine; ASPVL—Alginate:Span:PVA:Lidocaine; AGPVLAv—Alginate:Span:PVA:Aciclovir:Lidocaine; ASPVLAv—Alginate:Span:PVA:Lidocaine:Aciclovir.

**Table 2:** Thermogravimetric data for the decomposition stages of membranes.

Sample	Step	T_i_/°C	T_f_/°C	Δm %	Sample	Step	T_i_/°C	T_f_/°C	Δm %
AG	a b	27.09 116	115.56 267	10.478 63.459	AGPL	a b c	37.73 48.43 121.37	47.45 120.88 269.69	0.177 0.482 4.667
AS	a b c	39.3 191.36 330	109.47 329.64 405.23	6.575 45.904 15.919	ASPL	a b c d	38.64 50.28 194.49 297.05	49.83 194.94 296.6 406.32	2.055 13.297 31.277 14.991
AGAv	a b c d	39.16 50.23 132.95 199.49	49.33 131.6 199.49 279	2.939 6.741 23.962 30.316	AGPLAv	a b	37.43 69.09	68.64 406.96	3.062 74.763
ASAv	a b c d	39.06 50.2 196.73 277.79	50.2 194.95 275.57 405.62	3.284 9.138 33.102 15.734	ASPLAv	a b c d	38.53 52.46 202.56 299.64	50.66 200.77 297.39 405.7	1.285 9.674 28.284 20.420
AGL	a b c d e	39.15 55.74 135.55 194.74 271.86	53.05 134.21 194.23 271.85 405.47	2.554 6.923 18.931 35.530 7.139	AGPV	a b c d e	38.81 56.77 134.93 198.71 233.3	55.42 134.48 197.81 232.85 405.78	2.551 3.517 14.565 12.259 46.912
ASL	a b c d	39.32 52.71 169.59 273.54	52.26 168.7 272.65 405.59	1.683 9.277 43.376 11.798	ASPV	a b	39.67 200.97	199.03 382.12	4.811 64.088
AGLAv	a b c d e	30 39.17 122.54 197.84 282.11	54.41 122.09 196.95 281.21 405.37	3.290 5.875 18.804 35.205 8.385	AGPVAv	a b c d	37.23 115.45 202.42 253.43	115.45 201.93 252.46 405.99	7.644 19.387 48.338 13.683
ASLAv	a b c d	39.34 49.15 191.79 291.64	49.59 190.45 291.19 406.2	1.633 5.654 30.409 31.140	ASPVAv	a b c	39.49 196.48 258.5	193.09 257.05 406.78	4.636 10.395 51.273
AGP	a b c	37.75 54.95 145.03	53.14 145.03 284	3.094 7.642 66.342	AGPVL	a b c d	32.46 50.57 120.03 250.68	49.6 119.06 250.68 406.11	2.414 5.909 69.108 13.288
ASP	a b c	38 218.04 288.6	217.58 288.15 387.67	9.020 20.646 20.818	ASPVL	a b c	38.79 191.05 244.39	190.08 242.94 406.36	5.748 10.337 55.756
AGPAv	a b c	36.82 52.23 153.73	49.51 152.37 304.63	2.007 8.831 60.035	AGPVLAv	a b c d e	38.13 49.53 116.83 230.24 264.16	48.47 114.71 229.18 263.63 406.02	2.355 6.110 55.325 13.591 17.954
ASPAv	a b c	38.95 194.13 281.69	192.18 280.23 392.12	6.592 18.114 31.709	ASPVLAv	a b c	38.2 194.59 242.34	192.64 240.88 407.5	5.616 8.824 52.750

## Data Availability

The data presented in this study are available on request from the corresponding author.
